# Isolation of Asian endemic and livestock associated clones of methicillin resistant *Staphylococcus aureus* from ocular samples in Northeastern Iran

**Published:** 2013-09

**Authors:** Seyed Asghar Havaei, Amir Azimian, Hosein Fazeli, Mahmood Naderi, Kiarash Ghazvini, Siamak Mirab Samiee, Masoud Soleimani

**Affiliations:** 1 Department of Pathobiology, School of Medicine, Isfahan University of Medical Sciences, Isfahan, Iran; 2 Department of Pathobiology and Anatomy, School of Medicine, North Khorasan University of Medical Sciences, Bojnurd, Iran; 3 Molecular Biology and Genetic Engineering Department, Stem Cell Technology Research Center, Tehran, Iran; 4 Department of Microbiology, School of Medicine, Mashhad University of Medical Sciences, Mashhad, Iran; 5 Food and Drug Laboratory Research Center, Ministry of Health and Medical Education, No. 408, Emam Khomeini Ave., Tehran, Iran; 6 Department of Hematology, School of Medical Science, Tarbiat Modares University, Tehran, Iran

**Keywords:** *Staphylococcus aureus*, MRSA, MLST, *Spa* typing, *SCCmec* type, *agr*, ocular infection

## Abstract

**Background and Objectives:**

Methicillin Resistant *Staphylococcus aureus* (MRSA) strains are divided into Community Associated (CA-) and Hospital Associated (HA-) MRSA. These strains vary in antimicrobial resistance and pathogenicity. *S. aureus* is one of the most common microorganisms in ocular infections. This study was aimed to determine antimicrobial resistance patterns and genetic characteristics of MRSA strains isolated from ocular infections in Iran.

**Material and Methods:**

Out of 171 *S. aureus* strains isolated from various clinical samples during September-December 2011 at Mashhad Emam Reza Hospital, 3 were cultured from eye discharge samples. Antimicrobial resistance tests were performed with MIC and disk diffusion methods and also genetic evaluation was done with Staphylococcal Cassette Chromosome mec (*SCCmec)*, *Accessory Gene Regulator (agr*) and *Staphylococcal Protein A (spa)* typing, Multi Locus Sequence Typing (MLST) and determination of toxin gene profile.

**Results:**

All strains were MRSA and showed resistance to tetracycline, gentamicin and clindamycin too. Vancomycin, minocyclin and trimethoprim/sulfamethoxazole were effective on all ocular isolates. All isolates belonged to *SCCmec* IV type. MRSA1 belonged to ST239, CC8, *Spa* type t7688 and *agrIII* and had *tst1* and *hla* toxin genes. MRSA2 belonged to ST239, CC8, *Spa* type t037 and *agrI* and had the *hla* toxin gene. Finally, MRSA3 belonged to ST291, CC398, *Spa* type t304, and *agrI* and had *pvl* and *hla* toxin genes.

**Conclusion:**

Phenotypic and genotypic evaluation of the isolated MRSA strains revealed that these strains belong to endemic Asian and livestock related clones that could reach from other body sites or environment to the eye of patients and developed ocular infection.

## INTRODUCTION

*Staphylococcus aureus* is one of the major human pathogens causing a wide variety of infections from simple superficial skin infections to dangerous systemic infections such as septicemia, endocarditis and toxic shock syndrome (1, 2). *S. aureus* is the major ophthalmic bacterial pathogen isolated from various ocular infections such as conjunctivitis, blepharitis, external and internal hordeolum, scleritis, canaliculitis, keratitis (3, 4).

The eye is impermeable to external materials. In addition, some enzymes and immunomodulatory proteins such as lysozyme, lactoferrin, secretory immunoglobulins, and defensins are present in tear and reduce bacterial colonization in the ocular surfaces (5). Infections can originate from external sources or intraocular invasion of blood borne microorganisms. Bacterial infections on the outer surface of eye may spread to adjacent tissues such as the cornea, orbit, inner eye and even the brain (6). Major risk factors for bacterial ocular infections with external sources are surgical and non-surgical trauma and contact lens use. Ocular infections with progressive lesions can lead to blindness due to destructive activity of bacterial enzymes and toxins or even in some cases lead to mortality due to expansion of infection to other organs via the blood stream or the lymphatics (6, 7).

Treatment of *Staphylococcus aureus* infections has become more complicated with emergence of Methicillin-Resistant *Staphylococcus aureus* (MRSA) strain in 1961 (8, 9). MRSA was primarily a hospital-associated pathogen, however today multiple community acquired MRSA clones emerged in human infections. Recently, human-related MRSA infections based on their source, have been categorized into four groups; Hospital Associated MRSA (HA-MRSA), Community Associated MRSA (CA-MRSA), Hospital Associated MRSA with Community onset and Livestock Associated MRSA (LA-MRSA) (10). To date, all four types of MRSA strains have been isolated from clinical samples (10–15). HA-MRSA strains have more antimicrobial resistance and also cause more serious diseases mainly in hospitalized patients while CA-MRSA strains show less antimicrobial resistance and commonly lead to skin and soft tissue (SSI) infections (1).

Despite the fact that MRSA is one of the major topics in clinical microbiology research, very little is known about the occurrence and genetic characteristics of MRSA strains isolated from ocular infections in the world especially in Asian countries such as Iran. To the best of our knowledge, there is no report concerning the genetic characteristics of MRSA strains causing ocular infections in Iran. Here, we evaluated the genetic characteristics of three MRSAs isolated from ocular discharge samples of patients admitted to the Emam Reza Hospital, Mashhad, Khorasan Razavi, Iran.

## MATERIALS AND METHODS

171 Strains of *Staphylococcus aureus* were isolated from patients admitted between 23 September 2011 to 21 December 2011 at Emam Reza Hospital, a 918 bed University teaching Hospital, Mashhad, Iran. These isolates were obtained from blood, urine, sputum, wound, abscess, nose, throat, eye and respiratory tract samples. *S. aureus* isolates were identified by conventional biochemical tests including Gram staining, catalase, manitol fermentation, slide and tube coagulase test and DNase.

### Screening for methicillin and vancomycin resistance

All *S. aureus* isolates were screened for oxacillin and vancomycin resistance using agar screening method. Methicillin resistance was defined as the capability of growth in agar screening media including 4% NaCl plus 6µg/ml oxacillin and MIC ≥4 µg/ml whereas vancomycin resistance was defined as the capability of growth in agar screening media including 6 µg/ml vancomycin and MIC ≥16 µg/ml. Strains of *Staphylococcus aureus* ATCC 29213 and *Enterococcus faecalis* ATCC 52199 were used as control.

### Antimicrobial susceptibility testing

Antibiotic susceptibility testing was carried out using disk diffusion method (MAST DISKS™) according to guidelines of Clinical Laboratory Standards Institute (CLSI)(16, 17). The list of the antibiotics used in the test is presented in Table 2. Strain of *S. aureus* ATCC 25923 was used as control. E-Test method (Biomeriux Strips) was used for MIC determination for the only *S. aureus* strain capable of growing in the agar screening media.

### Genomic DNA extraction

Genomic DNA of *S. aureus* isolates were extracted using QIAamp^®^ DNA mini kit. According to manufacturer's protocol for bacterial cells, we added lysostaphin at the final concentration of 30µg/ml in lysis buffer.

### PCR

PCR amplification was performed with a TAKARA Gradient PCR TP600 thermal cycler in a volume of 50µl. We used EmeraldAmp^®^ MAX PCR Master Mix (Takara, Japan) for all PCR reactions.

### PCR identification of *mecA* gene

The primers used for amplification of *mecA* gene are listed in Table 3. PCR was performed with the following thermal setting: 5 min at 94°C for initial enzyme activation followed by 40 cycles of amplification (denaturation at 94°C for 30 sec; annealing at 57°C for 45 sec; extension at 72°C for 30 sec) and final extension at 72°C for 5 min.

### Multiplex PCR for detection of toxin genes

The primers used for amplification of *Panton-Valentine Leukocidin (pvl), Toxic Shock Syndrome Toxin-1 (tst1), alpha Hemolysin* (*hla*) and *Enterotoxin C* (*etc*) genes are listed in Table 3. PCR was performed with the following thermal setting: 5 min at 94°C for initial enzyme activation followed by 40 cycles of amplification (denaturation at 94°C for 40 sec; annealing at 60°C for 40 sec; extension at 72°C for 1 min) and final extension at 72°C for 5 min.

### Multiplex PCR for detection of toxin genes

The primers used for amplification of *Panton-Valentine Leukocidin (pvl), Toxic Shock Syndrome Toxin-1 (tst1), alpha Hemolysin* (*hla*) and *Enterotoxin C* (*etc*) genes are listed in Table 3****. PCR was performed with the following thermal setting: 5 min at 94°C for initial enzyme activation followed by 40 cycles of amplification (denaturation at 94°C for 40 sec**;** annealing at 60°C for 40 sec; extension at 72°C for 1 min) and final extension at 72°C for 5 min

### Multiplex PCR for *SCCmec* and *agr* typing

*SCCmec* and *agr* typing were performed as previously described (18, 19). The primers used for the PCR are listed in Table 3.

### MLST

Multi Locus Sequence Typing was carried out by PCR and sequencing of the internal fragments of carbamate kinase *(arc)*, shikimate dehydrogenase *(aro)*, glycerol kinase *(glp)*,guanylate kinase *(gmk)*, phosphate acetyl transferase *(pta)*, trios phosphate isomerase *(tpi)* and acetyl coenzyme A acetyl transferase *(yqi)* genes of *S. aureus* as previously described (20).

### *Spa* typing

*spa* typing was performed by PCR and sequencing of polymorphic X region of *spa* gene was done as previously described, http://spaserver.ridom.de/(21).

### Nucleotide Sequencing

Amplified PCR products were purified with QIAquick^®^ Gel Extraction Kit. The purified PCR products were sequenced with an ABI 3730XL DNA analyzer (Applied Biosystems) in both directions. The sequences were used for both confirmation and sequence based typing methods (MLST and *spa* typing).

## RESULTS

Of 171 *S. aureus* isolates 3 were isolated from eye discharge. These 3 isolates were resistant to oxacillin, positive for *mecA* gene and showed *SCCmec* type IV. They belonged to Clonal Complexes 8 and 398 (CC8 and CC398). Genetic analysis of the isolated MRSA strains revealed that these strains were endemic Asian and livestock related clones. All three isolates were sensitive to vancomycin, minocycline and trimethoprim/sulfamethoxazole but resistant to oxacillin, tetracycline, gentamycin and clindamycin. The demographic data and antibiogram results of these strains are listed in Table 1. Genetic evaluation results in details including, *mecA* gene PCR, SCC*mec* types, *agr* groups, toxin profiles, *spa* types and Sequence Types (STs) are presented in Table 2.

**Table 1:** Phenotypic characteristics of VISA isolates.

	Sex/ age	Sample/ ward	Oxa	Van	Min	Lev	Cip	Tet	Cot	Gen	Cli	Rif	Oxa MIC	Van MIC	Van Agar screen
**MRSA1**	F/25	Eye discharge/ out patient	R	S	S	R	R	R	S	R	R	S	128	2	-
**MRSA2**	F/39	Eye discharge/ out patient	R	S	S	R	R	R	S	R	R	S	128	1	-
**MRSA3**	F/50	Eye discharge / neurology	R	S	S	S	S	R	S	R	R	R	256	1	-

Abbreviations: Oxa: Oxacillin, Van: Vancomycin, Min: Minocyclin, Lev: Levofloxacin, Cip: Ciprofloxacin, Tet: Tetracycline, Cot: trimethoprim/sulfamethoxazole, Gen: Gentamycin, Cli: Clindamycin, Rif: Rifampicin. R: Resistant, S: Susceptible.

**Table 2:** Genetic characteristics of VISA isolates.

	*agr*	Scc *mec*	*pvl*	*hla*	*etc*	*tst1*	*mecA*	*vanA*	*spa* type	*spa* repeat profile	ST	CC	MLST allelic profile
**MRSA1**	III	IV	-	+	-	+	+	-	t7688	11-148-21-17-34-24-34-22-25	ST-239	8	2-3-1-1-4-4-3
**MRSA2**	I	IV	-	+	-	-	+	-	t037	15-12-16-02-25-17-24	ST-239	8	2-3-1-1-4-4-3
**MRSA3**	I	IV	+	+	-	-	+	-	t304	11-10-21-17-34-24-34-22-25	ST-291	398	3-37-19-2-20-26-32

Abbreviations: *pvl: Panton-ValentinLeukocidin, sec: Enterotoxin C, tst1: Toxic Shock Syndrome Toxin1, hla: alpha Hemolysin, agr: accessory gene regulator, SCCmec: Staphylococcal Cassette Chromosome,spa: Staphylococcal protein A*

**Table 3:** Primers used in this study.

Target	primer	Sequence (5’-3’)	Product size (bp)	reference
*mecA*	F R	AGAAGATGGTATGTGGAAGTTAG ATGTATGTGCGATTGTATTGC	583	
*pvl*	F R	GGAAACATTTATTCTGGCTATAC CTGGATTGAAGTTACCTCTGG	502	
*hla*	F R	CGGTACTACAGATATTGGAAGC TGGTAATCATCACGAACTCG	744	(24)
*sec*	F R	GGGAATGTTGGATGAAGG AGGCAAGCACCGAAGTAC	900	
*tst1*	F R	TTATCGTAAGCCCTTTGTTG TAAAGGTAGTTCTATTGGAGTAGG	398	
*agr*	Pan F R I R II R III R IV	ATGCACATGGTGCACATGC GTCACAAGTACTATAAGCTGCGAT GTATTACTAATTGAAAAGTGCCATAGC CTGTTGAAAAAGTCAACTAAAAGCTC CGATAATGCCGTAATACCCG	439 572 406 657	(27)
*SCCmec*	F β R α3 F ccrC R ccrC F 1272 R 1272 F 5R*mecA* R 5R431	ATTGCCTTGATAATAGCCYTCT TAAAGGCATCAATGCACAAACACT CGTCTATTACAAGATGTTAAGGATAAT CCTTTATAGACTGGATTATTCAAAATAT GCCACTCATAACATATGGAA CATCCGAGTGAAACCCAAA TATACCAAACCCGACAACTAC CGGCTACAGTGATAACATCC	937 518 415 359	(19)

## DISCUSSION

This is the first report of the molecular analysis of three *S. aureus* strains isolated from ocular infections with different molecular typing methods in Iran. In addition, *SCCmec* types of both isolated MRSA were type IV. With regard to these data, isolated strains can be categorized to CA-MRSA. The third MRSA was isolated from a hospitalized patient who had a history of hospitalization. Given this information, this infection could be categorized into the Hospital associated group, however, the isolate showed *SCCmec* type IV and belonged to ST291 (CC398) and therefore can be placed in the LA-MRSA group. We concluded that this isolate was a livestock associated strain that either entered the hospital environment or colonized the patient before hospitalization. We don't know about the patient contact with livestock or animals.

Nadig *et al*. (2012) reported that most of the eye infections in two important Eye Specialist Hospital in India were caused by community-associated *S. aureus*. They also reported that major Sequence Type in their isolates was ST722 (belonged to Clonal Complex 1) whereas in our study isolated strains were belonged to endemic Asian and also livestock associated clones (10). The livestock associated isolate in our study epidemiologically was HA-MRSA. Currently, MRSA isolates belonging to CC398, such as ST398 or ST291 (Double Locus Variant of ST398) are newly categorized to LA-MRSA (10). There are some reports relating human infections, caused by Livestock associated Methicillin Sensitive *Staphylococcus aureus* (MSSA), to LA-MRSA (13–15). To our knowledge, this is the first report of human ocular infection with LA-MRSA. Mediavilla *et al*. reported that most of CC398 strains isolated from Clinical samples in the USA, mainly belonged to *Spa* type t2313 which is endemic to Europe, while our LA-MRSA isolate had *Spa* type t304 that has been isolated from different parts of the world (13, 22).

The MRSA1 strain belonged to *Spa* type t7688 and MRSA2 to *Spa* type t037. To date, *Spa* type t7688 has been reported for the first time from Iran (http://spa.ridom.de/spa-t7688.shtml) and t037 is one of the common *Spa* types in Asian countries (23, 24). Hesje *et al*. reported that the most observed *Spa* types in their study in the USA were *Spa* types t008 and t002 that belonged to USA300 and USA100 clones, respectively (25).

Other important genetic characteristic of these isolates is *agr* specific group. The *agr* locus of *S. aureus* is a quorum-sensing gene cluster that up-regulates the production of secreted virulence factors and down-regulates the production of cell-associated virulence factors. The relation between *agr* group, type of infection, geographical area and resistance to some antibiotics, particularly vancomycin, is highlighted in some articles (18, 26). MRSA1 belonged to *agrIII* and MRSA2 & 3 belonged to *agrI*, that is the most common *agr* group among worldwide MRSA clones (27).

Some reports have been published concerning the antimicrobial susceptibility patterns of MRSA strains isolated from clinical specimens including ocular samples (5–7, 28–32). Shanmuganathan *et al*. reported that all MRSA strains, isolated from external ocular infections, were sensitive to chloramphenicol and those isolated from patients more than 50 years old, all were resistant to ofloxacin (28). Hsiao *et al*. reported that CA-MRSA and HA-MRSA isolates were resistant to clindamycin and erythromycin and CA-MRSA isolates were more susceptible to trimethoprim/sulfamethoxazole (30). All MRSA strains in this study were resistant to tetracycline, gentamicin and clindamycin. In contrast, vancomycin, minocyclin and sulfamethoxazole/trimethoprim showed activity against all of the isolated MRSA strains (Table 2).

Epidemiological studies show that combination of *pvl* and other toxins such as entero-toxins, could increase the severity of diseases caused by *S. aureus* (6). We evaluated *pvl*, *tst1*, *etc* and *hla* genes amongst the MRSA isolate (Table 1). All isolates were positive for *hla* gene and negative for *etc* gene. *hla* gene commonly found in most isolated *S. aureus* strains (33). MRSA1 was also positive for *tst1* in PCR and MRSA3 isolate was positive for *pvl* gene, but unfortunately we do not have any data about the outcome of these infections in our patients, because they were not available for more questioning.

A major limitation in this study was the small number of isolates. It was better that we simultaneously evaluated the nasal swab and eye samples. This approach helped us to find probable source of ocular infection in the patients. An interesting finding of the ocular specimens in this study was the gender of patients: all samples were obtained from female patients. However, due to small sample size it is not possible to make conclusion about this finding.

### Funding

This work was supported bythe Isfahan University of Medical Sciences [Grant number 390256].
